# Ni^II^ mol­ecular complex with a tetra­dentate amino­guanidine-derived Schiff base ligand: structural, spectroscopic and electrochemical studies and photoelectric response

**DOI:** 10.1107/S2056989022000317

**Published:** 2022-01-14

**Authors:** Olga Yu. Vassilyeva, Elena A. Buvaylo, Vladimir N. Kokozay, Sergey L. Studzinsky, Brian W. Skelton, Georgii S. Vasyliev

**Affiliations:** a Department of Chemistry, Taras Shevchenko National University of Kyiv, 64/13 Volodymyrska Street, Kyiv 01601, Ukraine; b School of Molecular Sciences, M310, University of Western Australia, Perth, WA 6009, Australia; c National Technical University of Ukraine "Igor Sikorsky Kyiv Polytechnic Institute", 37 Prospect Peremohy, Kyiv 03056, Ukraine

**Keywords:** crystal structure, amino­guanidine, Schiff base ligand, square-planar Ni^II^ complex

## Abstract

The new mol­ecular nickel(II) complex, namely, {4-bromo-2-[({*N*′-[(2-oxidobenzylidene)amino]carbamimidoyl}imino)methyl]phenolato}nickel(II) *N*,*N*-di­methyl­formamide solvate monohydrate, [Ni(C_15_H_11_BrN_4_O_2_)]·C_3_H_7_NO·H_2_O, (I), crystallizes in the triclinic space group *P*  with one mol­ecule per asymmetric unit. The guanidine ligand is a product of Schiff base condensation between amino­guanidine, salicyl­aldehyde and 5-bromo­salicyl­aldehyde templated by Ni^2+^ ions. The chelating ligand mol­ecule is deprotonated at the phenol O atoms and coordinates the metal centre through the two azomethine N and two phenolate O atoms in a *cis*-NiN_2_O_2_ square-planar configuration [average(Ni—N/O) = 1.8489 Å, *cis* angles in the range 83.08 (5)–95.35 (5)°, *trans* angles of 177.80 (5) and 178.29 (5)°]. The complex mol­ecule adopts an almost planar conformation. In the crystal, a complicated hydrogen-bonded network is formed through N—H⋯N/O and O—H⋯O inter­molecular inter­actions. Complex (I) was also characterized by FT–IR and ^1^H NMR spectroscopy. It undergoes an Ni^II^ ↔ Ni^III^ redox reaction at *E*_1/2_ = +0.295 V (*vs* Ag/AgCl) in methanol solution. In a thin film with a free surface, complex (I) shows a fast photoelectric response upon exposure to visible light with a maximum photovoltage of ∼178 mV.

## Abstract 2

Ni^II^ ions templated the condensation of amino­guanidine with two different aldehyde mol­ecules with the formation of a new mol­ecular nickel(II) complex with a tetra­dentate chelating ligand.

## Chemical context

Guanidine, the functional group on the side chain of arginine, has attracted much attention in the fields of drug development (Santos *et al.*, 2015 ▸; Hirsh *et al.*, 2008 ▸) and natural product synthesis (Berlinck & Romminger, 2016 ▸; Kudo *et al.*, 2016 ▸). Guanidine derivatives have also been explored as catalysts and superbases (Selig, 2013 ▸; Ishikawa, 2009 ▸). Amino­guanidine (AG) is an anti­oxidant and nucleophilic agent with strong scavenging activities against reactive carbonyl species (RCS) – a class of byproducts originating from exogenous and endogenous oxidation. RCS react with nucleophilic targets such as nucleic acids, phospho­lipids and proteins to form damaging adducts (Colzani *et al.*, 2016 ▸; Ramis *et al.*, 2019 ▸). Diabetic and Alzheimer’s disease patients were both found to have increased RCS levels in their circulatory systems (Kalousova *et al.*, 2002 ▸; Picklo *et al.*, 2002 ▸). Blocking RCS by carbonyl quenchers is an encouraging therapeutic strategy and the investigation of conjugates of AG and aryl­aldehydes as well as their metal complexes has been at the focus of research inter­est for several decades (Fukumoto *et al.*, 2002 ▸; Qian *et al.*, 2010 ▸; Vojinović-Ješić *et al.*, 2014 ▸). 
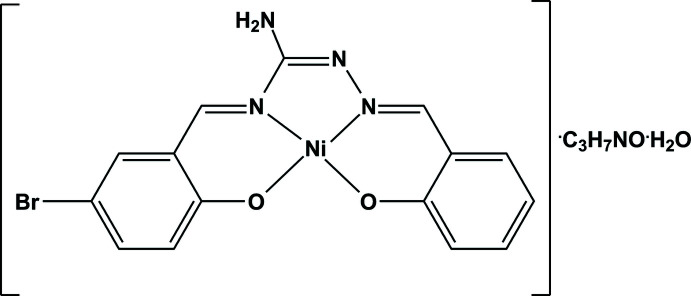


In our previous study, the condensation reactions of amino­guanidine freshly liberated from AG·HCl or AG·HNO_3_ and aryl­aldehydes (salicyl­aldehyde, 5-bromo­salicyl­aldehyde, pyridine-2-carbaldehyde) produced the expected 1:1 Schiff base ligands isolated as protonated cations of nitrate or chloride salts as well as Cu^II^ and Co^III^ mononuclear complexes (Buvaylo *et al.*, 2013 ▸, 2016 ▸, 2017 ▸). The di­chlorido­copper(II) complex bearing a pyridine-2-carbaldehyde amino­guanidine Schiff base ligand revealed prominent catalytic activity towards the oxidation of cyclo­hexane with hydrogen peroxide in the presence of various promoters (Buvaylo *et al.*, 2017 ▸). In contrast, the inter­action of AG with formaldehyde yielded a completely different compound with a high nitro­gen content that had not been reported before (Buvaylo *et al.*, 2018 ▸). 2,20-Methyl­enedihydrazinecarboximidamide, which was isolated in its protonated form as the dinitrate salt, resulted from the condensation between two AG mol­ecules and one mol­ecule of formaldehyde.

In the present work, we attempted to synthesize an Ni complex with the Schiff base ligand derived from AG and salicyl­aldehyde. However, 5-bromo­salicyl­aldehyde was also mistakenly introduced into the flask. As a result, the new tetra­dentate ligand (2-hy­droxy­benzyl­idene)(5-bromo-2-hy­droxy­benzyl­idene)amino­guanidine, H_2_ *L*, was formed from the *in situ* condensation of one AG mol­ecule and two different mol­ecules of the aldehydes in the presence of Ni^2+^ ions. Herein, the crystal structure of [Ni*L*]·DMF·H_2_O (DMF = *N*,*N*-di­methyl­formamide), (I), is presented along with the elemental analyses, IR, NMR and cyclic voltammetry results as well as photoelectric response characteristics.

## Structural commentary

**Figure 1:**
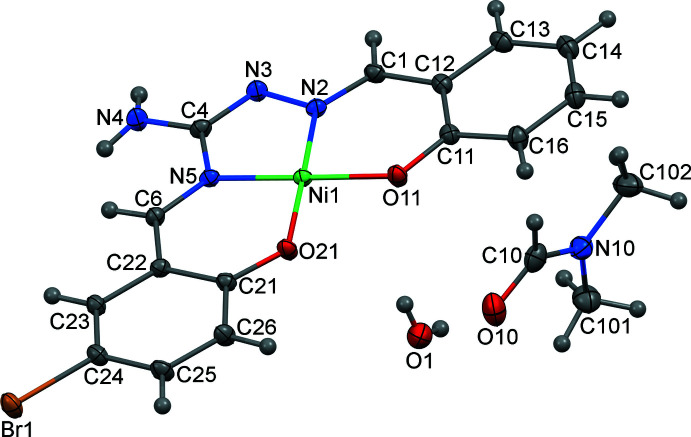
Mol­ecular structure and atom labelling of [Ni*L*]·C_3_H_7_NO·H_2_O (I), with displacement ellipsoids at the 50% probability level.

Compound (I), [Ni(C_15_H_11_BrN_4_O_2_)]·C_3_H_7_NO·H_2_O, crystallizes in the triclinic space group *P* 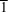 and is assembled from discrete Ni*L* mol­ecules and solvent mol­ecules of crystallization. The chelating ligand *L* ^2–^ is deprotonated at the phenol O atoms and coordinates the Ni^II^ ion through the two azomethine N and two phenolate O atoms in a *cis*-NiN_2_O_2_ square-planar configuration (Fig. 1 ▸). The Ni—N/O distances fall in the range 1.8383 (11)–1.8562 (10) Å, the *cis* angles at the metal atom vary from 83.08 (5) to 95.35 (5)° and the *trans* angles are equal to 177.80 (5) and 178.29 (5)° (Table 1 ▸). The mol­ecule is quite planar, the atoms with the largest deviations being C15 [δ = 0.059 (2) Å] and C23 [δ = 0.057 (2) Å] although there is very slight ‘bowing’ at the Ni atom. The dihedral angle between the two phenyl rings is 3.37 (5)°.

## Supra­molecular features

**Figure 2:**
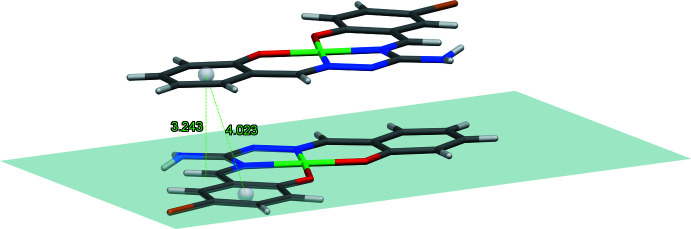
View of a pair of centrosymmetically related *trans*-oriented Ni*L* mol­ecules showing the absence of π–π stacking.

**Figure 3:**
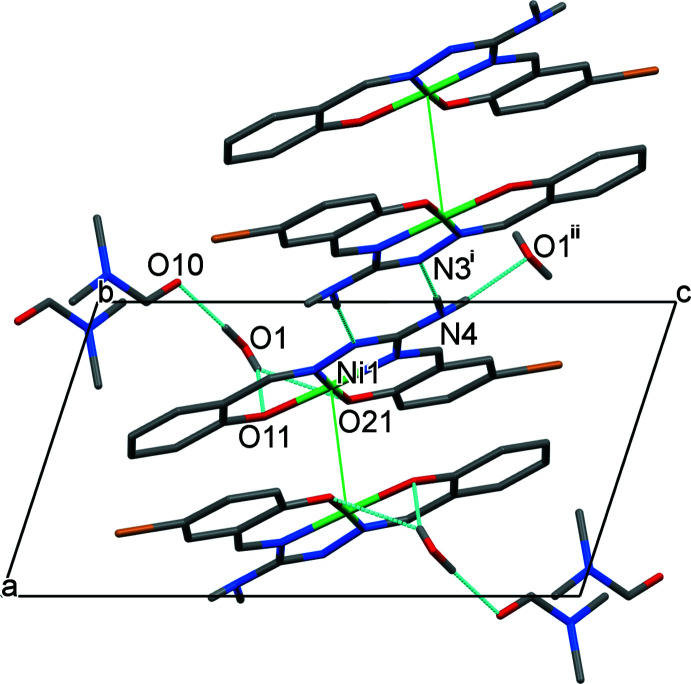
Fragment of the crystal packing of (I), viewed along the *b*-axis direction, showing inter­molecular N—H⋯N/O and O—H⋯O inter­actions (CH hydrogen atoms were omitted for clarity; hydrogen bonds are shown as blue dashed lines; green lines joining Ni centres do not represent bonds). [Symmetry codes: (i) −*x* + 2, −*y* + 2, −*z* + 1; (ii) −*x* + 2, −*y* + 1, −*z* + 1.]

In the crystal, the Ni*L* mol­ecules form centrosymmetrically related pairs with an inter­planar distance of approximately 3.32 Å and the Ni⋯Ni separation being 3.4191 (3) Å (Fig. 2 ▸). There are no hydrogen bonds between the Ni*L* mol­ecules and no π–π stacking is observed owing to the *trans*-orientation of the two paired mol­ecules. Instead, the Ni*L* mol­ecule creates centrosymmetric hydrogen-bonded pairs through one H atom on the amine nitro­gen N4, its other hydrogen forming a hydrogen bond to a centrosymmetrically related water mol­ecule as shown by the N4⋯N3 {−*x* + 2, −*y* + 2, −*z* + 1} and N4⋯O1 {−*x* + 2, −*y* + 1, −*z* + 1} distances of 3.0116 (17) and 2.8900 (19) Å, respectively (Fig. 3 ▸, Table 2 ▸). One hydrogen atom of the solvent water mol­ecule is involved in a bifurcated hydrogen bond to the two coordinated phenolate oxygen atoms, O11 and O21, with corresponding O⋯O distances of 3.0056 (17) and 3.0719 (18) Å, respectively. The other hydrogen atom of the water mol­ecule makes a hydrogen bond to the DMF oxygen atom O10 with the O1⋯O10 distance being equal to 2.772 (2) Å. This forms a three-dimensional hydrogen bonded network.

## Database survey

Crystal structures of neither the ligand itself nor its metal complexes are found in the Cambridge Structure Database (CSD, Version 5.42, update of May 2021; Groom *et al.*, 2016 ▸). AG tends to inter­act with aldehyde groups in the familiar and important amine–aldehyde condensation reaction in a 1:1 molar ratio. The structures of 45 of this kind of AG-based Schiff bases and their metal complexes deposited in the CSD incorporate various derivatives of benzaldehyde, pyridine and pyrimidine. Most of the Schiff base metal complexes derived from AG are mononuclear with the ligands coordinating through two azomethine N atoms and phenolate O atom from the ring if such a one is present. Schiff base condensations with molar ratios different from 1:1 usually employ AG amino derivatives, such as *e.g.* tri­amino­guanidine. The product of the 1:3 condensation reaction of the latter and 5-bromo­salicyl­aldehyde, the tris­[(5-bromo-2-hy­droxy­benzyl­idene)amino]­guanidinium cation was found suitable for coordination of three Cd^2+^ centres to form chiral (although racemic), tightly closed tetra­hedral cages with a formal [*M*_6_ *L*_4_] topology, where *M* is a (CdO)_2_ four-membered ring (FIKJIT, FIKJOZ, FIKJUF; Müller *et al.*, 2005 ▸).

To our knowledge, only one example of a Schiff base metal complex structurally similar to (I) has been reported. The reaction between (salicyl­idene­amino)­nitro­guanidine and salicyl­aldehyde in the presence of Ni^2+^ ions used as templating agents and K^+^ cations produced potassium (*N*,*N′*-bis­(salicyl­idene­amino)-*N′′*-nitro­guanidinato-*N*,*N′*,*O*,*O′*)nickel(II) with a *cis*-NiN_2_O_2_ square-planar chromophore (TUFDAZ; Starikova *et al.*, 1996 ▸). Obviously, the Ni^II^-assisted condensation of AG or its NO_2_-substituted analogue with two aldehyde mol­ecules in the case of (I) and TUFDAZ occurred due to a combination of structural and electronic factors unique to the nickel(II) cation, which is prone to adopt a tetra­dentate square-planar geometry, and the favourable stoichiometry of the condensation reaction.

## IR and ^1^H NMR spectroscopy measurements

The infrared spectrum of complex (I) in the 4000–400 cm^−1^ range is very rich and shows all characteristic functional group peaks. A broad absorption near 3500 cm^−1^ and multiple overlapping bands in the range 3358–3134 cm^−1^ are attributed to ν(OH) and ν(NH) stretching vibrations, respectively. Bands arising above 3000 cm^−1^ are due to aromatic =CH stretching of the ligand; alkyl CH stretching vibrations of *L* ^2–^ and DMF solvent are seen from 2958 to 2808 cm^−1^. Very intense overlapping signals in the 1668–1584 cm^−1^ region represent ν(C=O) stretching of the DMF mol­ecule, deformation vibrations of the amino group, a group mode of the CN_3_ unit of the ligand, ν_as_(CN_3_), and ν(C=N) peaks of *L* ^2–^ that cannot be distinguished from each other. The symmetric stretching mode ν_s_(CN_3_) of the CN_3_ unit falls in the 1600–1400 cm^−1^ range of the aromatic ring vibrations. Several sharp bands of medium intensity are observed in the out-of-plane CH bending region (800–700 cm^−1^).

**Figure 4:**
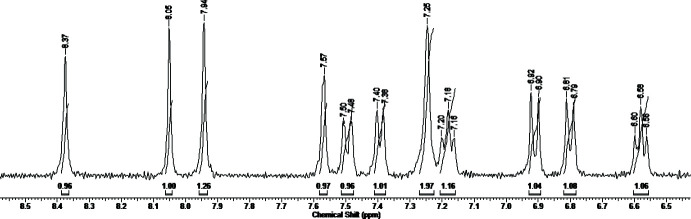
400 MHz ^1^H NMR spectrum of (I) in DMSO-*d*_6_ at 293 K in the range 8.5–6.5 ppm.

The diamagnetic nature of the majority of square-planar Ni^II^ complexes is helpful in their characterization by NMR spectroscopy. The ^1^H NMR spectrum of (I) exhibits the expected set of signals between 8.5 and 2.5 ppm (Fig. 4 ▸). The presence of two –CH=N– protons that appear as two singlets in a 1:1 ratio at δ 8.37 and 8.05 ppm confirms the Schiff base condensation of AG with two aldehyde mol­ecules. The signals of seven aromatic protons in the range 7.57–6.58 ppm observed as one singlet, four doublets and two triplets evidence the presence of two chemically inequivalent rings. A broad singlet at δ 7.25 ppm is due to the NH_2_ group adjacent to the carbon atom of the guanidine moiety. The absence of the phenolic OH singlets detected at δ 11.55 ppm in the ^1^H NMR spectrum of (5-bromo­salicyl­idene)amino­guanidine·HNO_3_ (Buvaylo *et al.*, 2016 ▸) points out the deprotonation of H_2_ *L* upon coordination to the Ni^II^ centre in (I). Three sharp singlets in a 1:3:3 ratio at 7.94, 2.88 and 2.72 ppm were attributed to the CH and two CH_3_ groups of DMF, respectively.

## Cyclic voltammetry

**Figure 5:**
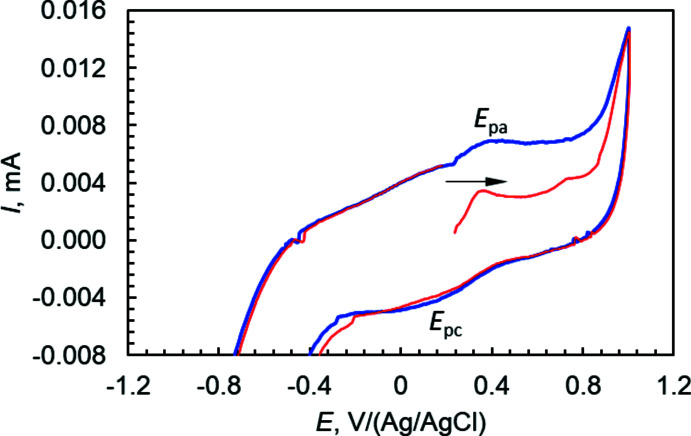
Cyclic voltammogram of (I), 0.1 m*M* in methanol mixed with 0.1 *M* acetate buffer (pH 4) and NaClO_4_ (70:28:2) as supporting electrolyte at a glassy carbon electrode and Ag/AgCl as a reference electrode (scan rate: 100 mV s^−1^; *T* = 293 K).

The electrochemical features of complex (I) were studied in methanol in the presence of 0.1 *M* acetate buffer (pH 4) and NaClO_4_ (70:28:2) as supporting electrolyte by using a three-electrode setup (glassy carbon working electrode, platinum auxiliary electrode and Ag/AgCl reference electrode) in the potential range +1.0 to −1.0 V at a scan rate of 100 mV s^−1^. The anodic scan, starting from the open circuit potential (0.24 V vs Ag/AgCl), displays an oxidation wave at *E*_pa_ = +0.42 V coupled with a corresponding reduction wave at *E*_pc_ = +0.17 V (Fig. 5 ▸). A large separation between the cathodic and anodic peak potentials (250 mV) indicates a quasi-reversible redox process which can be assigned to Ni^+2^/Ni^+3^ couple with *E*_1/2_ = +0.295 V (vs Ag/AgCl). The non-equivalent current intensity of cathodic and anodic peaks (*i*_c_/*i*_a_ = 0.551) suggests that the Ni^III^ complex generated by oxidation of Ni^II^ is not stable.

## Electro-optical measurements

The ability of (I) to form thin films on its own when cast from methanol solution prompted us to examine its photoelectric response under illumination with visible light. The thin film of the complex with estimated thickness of about 1.5 µm was obtained by drop casting of a methanol solution of (I) on an electroconducting ITO (SnO_2_: In_2_O_3_) layer of a standard glass slide and subsequent drying. A Kelvin probe technique was employed to track the contact potential difference between the free surface of the film and the probe with a BM8020 USB oscilloscope according to Davidenko *et al.* (2016 ▸). A 4 mm diameter aluminium plate placed ∼50 µm above the surface with a vibration frequency of 4 kHz was used as the reference probe. A white-light-emitting diode (LED) with power density *I* ≃ 40 W m^−2^ was used to illuminate the film from the ITO substrate side.

**Figure 6:**
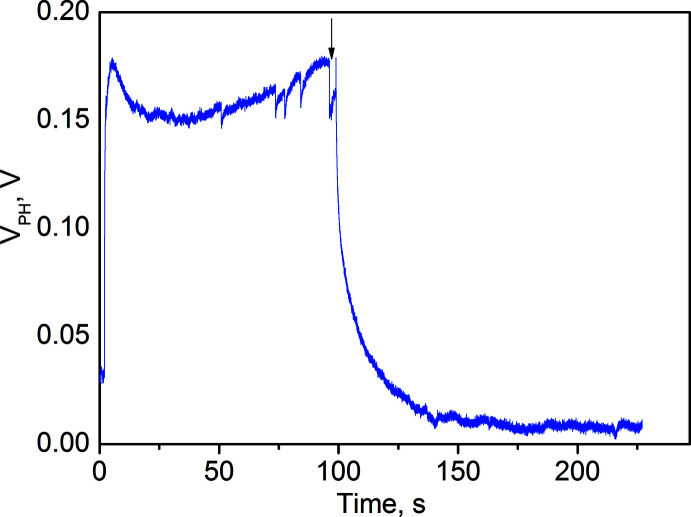
Time dependence of *V*_PH_ of a thin film sample of (I) with a free film surface upon illumination with a white LED (*I* = 40 W m^−2^) from the side of a transparent ITO electrode; illumination stopped at the point shown by the vertical arrow.

The thin-film sample of (I) showed a rather fast photoelectric response upon exposure to visible light with the surface potential V_PH_ reaching its maximum value of ∼178 mV within 6 s. Then the potential diminished slightly to stay nearly constant until the light was turned off at *t* = 100 s (Fig. 6 ▸). The V_PH_ relaxation in the film occurred almost as fast as its growth. The free surface of the film acquired a positive charge under illumination meaning the photogenerated electrons transfer to the ITO substrate. The fast kinetics of the surface photovoltage growth and decay indicates a high mobility of the photogenerated charge carriers in (I).

## Synthesis and crystallization

A mixture of salicyl­aldehyde (0.20 g, 2 mmol), 5-bromo­salicyl­aldehyde (0.40 g, 2 mmol), AG·HCl (0.22 g, 2 mmol) and NiCl_2_·6H_2_O (0.24 g, 1 mmol) in DMF (5 mL) in a conical flask was heated at 323 K under magnetic stirring for 1.5 h with its green colour deepening. Then the solution was filtered and allowed to stand at room temperature. It changed colour to brown upon filtration. After a week, diethyl ether (2 mL) was added to the clear solution to initiate precipitation. Red shiny plate-like crystals of the title compound formed over a month. They were filtered off, washed with diethyl ether and dried out in air (yield based on NiCl_2_·6H_2_O: 69%). Analysis calculated for C_18_H_20_BrN_5_NiO_4_ (509.01): C, 42.48; H, 3.96; N, 13.76%. Found: C, 42.55; H, 3.74; N, 13.70%. ^1^H NMR (400 MHz, DMSO-*d*_6_, *s*, singlet; *br*, broad; *d*, doublet; *t*, triplet): δ 8.37, 8.05 (*s*, 2H, 2 × CH=N); 7.94 (*s*, 1H, CH_DMF_); 7.57 (*s*, 1H, ring); 7.50 (*d*, 1H, ring, *J* = 9.0 Hz); 7.39 (*d*, 1H, ring, *J* = 8.0 Hz); 7.25 (*br*, 2H, NH_2_); 7.18 (*t*, 1H, ring, *J* = 7.0 Hz); 6.91 (*d*, 1H, ring, *J* = 10.0 Hz), 6.80 (*d*, 1H, ring, *J* = 8.5 Hz), 6.58 (*t*, 1H, ring, *J* = 7.4 Hz); 2.88, 2.72 [*s*, 6H, 2 × CH_3_(DMF)]. FT–IR (KBr, ν cm^−1^): 3502*br*, 3358*m*, 3278*m*, 3248*m*, 3134*m*, 3062*m*, 2958*w*, 2930*w*, 2884*w*, 2832*w*, 2808*w*, 1668*vs*, 1610*vs*, 1584*s*, 1536*w*, 1512*m*, 1452*s*, 1412*m*, 1384*m*, 1356*m*, 1310*m*, 1246*w*, 1206*m*, 1184*m*, 1152*w*, 1106*w*, 1066*w*, 990*w*, 948*w*, 908*w*, 826*w*, 754*m*, 690*w*, 668*w*, 656*w*, 616*w*, 582*w*, 550*w*, 532*w*, 462*w*, 448*w*, 410*w*.

## Refinement

Crystal data, data collection and structure refinement details are summarized in Table 3 ▸. All hydrogen atoms bound to carbon were included in calculated positions and refined using a riding model with isotropic displacement parameters based on those of the parent atom (C—H = 0.95 Å, *U*_iso_(H) = 1.2*U*_eq_C for CH, C—H = 0.98 Å, *U*_iso_(H) = 1.5*U*_eq_C for CH_3_). Water and NH_2_ hydrogen atoms were refined without restraints. Anisotropic displacement parameters were employed for the non-hydrogen atoms.

## Supplementary Material

Crystal structure: contains datablock(s) I, global. DOI: 10.1107/S2056989022000317/dj2041sup1.cif

Structure factors: contains datablock(s) I. DOI: 10.1107/S2056989022000317/dj2041Isup2.hkl

CCDC reference: 1958727

Additional supporting information:  crystallographic information; 3D view; checkCIF report

## Figures and Tables

**Table 1:** Selected geometric parameters (Å, °)

Ni1—N2	1.8383 (11)	Ni1—O21	1.8515 (10)
Ni1—N5	1.8494 (11)	Ni1—O11	1.8562 (10)
			
N2—Ni1—N5	83.08 (5)	N2—Ni1—O11	95.35 (5)
N2—Ni1—O21	177.80 (5)	N5—Ni1—O11	178.29 (5)
N5—Ni1—O21	95.25 (5)	O21—Ni1—O11	86.30 (4)

**Table 2:** Hydrogen-bond geometry (Å, °)

*D*—H⋯*A*	*D*—H	H⋯*A*	*D*⋯*A*	*D*—H⋯*A*
N4—H4*A*⋯N3^i^	0.85 (2)	2.16 (2)	3.0116 (17)	176 (2)
N4—H4*B*⋯O1^ii^	0.81 (2)	2.09 (2)	2.8900 (19)	169 (2)
O1—H1*A*⋯O11	0.72 (3)	2.38 (3)	3.0056 (17)	146 (3)
O1—H1*A*⋯O21	0.72 (3)	2.48 (3)	3.0719 (18)	141 (3)
O1—H1*B*⋯O10	0.80 (3)	1.97 (3)	2.772 (2)	178 (3)

Symmetry codes: (i) -x+2, -y+2, -z+1; (ii) -x+2, -y+1, -z+1.

**Table 3:** Experimental details

Crystal data	
Chemical formula	[Ni(C_15_H_11_BrN_4_O_2_)]·C_3_H_7_NO·H_2_O
*M*_r_	509.01
Crystal system, space group	Triclinic, *P*\overline{1}
Temperature (K)	100
*a*, *b*, *c* (Å)	8.3057 (4), 9.2300 (4), 14.3970 (7)
α, β, γ (°)	95.338 (4), 104.493 (4), 112.592 (5)
*V* (Å^3^)	964.23 (9)
*Z*	2
Radiation type	Mo *K*α
μ (mm^−1^)	3.12
Crystal size (mm)	0.32 × 0.26 × 0.12

Data collection	
Diffractometer	Oxford Diffraction Xcalibur diffractometer
Absorption correction	Analytical (*CrysAlis PRO*; Rigaku OD, 2016 ▸)
*T*_min_, *T*_max_	0.484, 0.721
No. of measured, independent and observed [*I* > 2σ(*I*)] reflections	28563, 9442, 7711
*R*_int_	0.033
(sin θ/λ)_max_ (Å^−1^)	0.837

Refinement	
*R*[*F* ^2^ > 2σ(*F* ^2^)], *wR*(*F* ^2^), *S*	0.036, 0.083, 1.04
No. of reflections	9442
No. of parameters	280
H-atom treatment	H atoms treated by a mixture of independent and constrained refinement
Δρ_max_, Δρ_min_ (e Å^−3^)	0.73, −0.39

Computer programs: *CrysAlis PRO* (Rigaku OD, 2016 ▸), *SHELXT* (Sheldrick, 2015*a* ▸), *SHELXL2017* (Sheldrick, 2015*b* ▸), *Mercury* (Macrae *et al.*, 2020 ▸) and *WinGX* (Farrugia, 2012 ▸).

## supplementary crystallographic information

### Crystal data

**Table:**

[Ni(C_15_H_11_BrN_4_O_2_)]·C_3_H_7_NO·H_2_O	*Z* = 2
*M**_r_* = 509.01	*F*(000) = 516
Triclinic, *P*1	*D*_x_ = 1.753 Mg m^−^^3^
Hall symbol: -P 1	Mo *K*α radiation, λ = 0.71073 Å
*a* = 8.3057 (4) Å	Cell parameters from 11025 reflections
*b* = 9.2300 (4) Å	θ = 3.4–37.3°
*c* = 14.3970 (7) Å	µ = 3.12 mm^−^^1^
α = 95.338 (4)°	*T* = 100 K
β = 104.493 (4)°	Plate, red
γ = 112.592 (5)°	0.32 × 0.26 × 0.12 mm
*V* = 964.23 (9) Å^3^	

### Data collection

**Table:**

Oxford Diffraction Xcalibur diffractometer	9442 independent reflections
Graphite monochromator	7711 reflections with *I* > 2σ(*I*)
Detector resolution: 16.0009 pixels mm^-1^	*R*_int_ = 0.033
ω scans	θ_max_ = 36.5°, θ_min_ = 3.4°
Absorption correction: analytical (CrysAlis Pro; Rigaku OD, 2016)	*h* = −13→13
*T*_min_ = 0.484, *T*_max_ = 0.721	*k* = −15→15
28563 measured reflections	*l* = −23→24

### Refinement

**Table:**

Refinement on *F*^2^	Primary atom site location: dual
Least-squares matrix: full	Hydrogen site location: mixed
*R*[*F*^2^ > 2σ(*F*^2^)] = 0.036	H atoms treated by a mixture of independent and constrained refinement
*wR*(*F*^2^) = 0.083	*w* = 1/[σ^2^(*F*_o_^2^) + (0.0353*P*)^2^ + 0.1785*P*] where *P* = (*F*_o_^2^ + 2*F*_c_^2^)/3
*S* = 1.04	(Δ/σ)_max_ = 0.002
9442 reflections	Δρ_max_ = 0.73 e Å^−^^3^
280 parameters	Δρ_min_ = −0.39 e Å^−^^3^
0 restraints	

### Special details

**Table:**

Geometry. All esds (except the esd in the dihedral angle between two l.s. planes) are estimated using the full covariance matrix. The cell esds are taken into account individually in the estimation of esds in distances, angles and torsion angles; correlations between esds in cell parameters are only used when they are defined by crystal symmetry. An approximate (isotropic) treatment of cell esds is used for estimating esds involving l.s. planes.
Refinement. Water molecule and NH_2_ hydrogen atoms were refined without restraints.

### Fractional atomic coordinates and isotropic or equivalent isotropic displacement parameters (Å^2^)

**Table:**

	*x*	*y*	*z*	*U*_iso_*/*U*_eq_	
Ni1	0.71284 (2)	0.50588 (2)	0.54926 (2)	0.01171 (4)	
Br1	0.78673 (2)	−0.02916 (2)	0.16762 (2)	0.02100 (4)	
C11	0.59223 (17)	0.53634 (16)	0.71522 (10)	0.0144 (2)	
O11	0.61079 (14)	0.44311 (12)	0.64783 (8)	0.01633 (18)	
C12	0.65390 (17)	0.70528 (16)	0.72467 (10)	0.0145 (2)	
C13	0.62837 (19)	0.79600 (18)	0.79975 (11)	0.0183 (3)	
H13	0.670747	0.908793	0.805476	0.022*	
C14	0.5434 (2)	0.72456 (19)	0.86487 (11)	0.0203 (3)	
H14	0.528595	0.787163	0.915561	0.024*	
C15	0.4794 (2)	0.55819 (19)	0.85484 (11)	0.0205 (3)	
H15	0.418098	0.507219	0.898316	0.025*	
C16	0.5039 (2)	0.46680 (18)	0.78260 (11)	0.0182 (3)	
H16	0.460325	0.354169	0.778076	0.022*	
C1	0.73956 (18)	0.78904 (17)	0.65882 (10)	0.0150 (2)	
H1	0.776971	0.901853	0.668905	0.018*	
N2	0.76894 (15)	0.71997 (13)	0.58640 (9)	0.01352 (19)	
N3	0.85703 (16)	0.82274 (14)	0.53203 (9)	0.0156 (2)	
C4	0.88158 (18)	0.74226 (16)	0.46136 (10)	0.0142 (2)	
N4	0.96020 (18)	0.81776 (15)	0.39828 (10)	0.0179 (2)	
N5	0.81845 (15)	0.57454 (13)	0.45334 (8)	0.01256 (19)	
C6	0.83447 (17)	0.48570 (16)	0.38291 (10)	0.0134 (2)	
H6	0.887782	0.537164	0.336972	0.016*	
C21	0.69562 (17)	0.22941 (16)	0.43607 (10)	0.0135 (2)	
O21	0.66497 (14)	0.29247 (12)	0.51103 (8)	0.01554 (18)	
C22	0.77713 (17)	0.31744 (15)	0.37102 (10)	0.0129 (2)	
C23	0.80347 (18)	0.23840 (16)	0.29043 (10)	0.0145 (2)	
H23	0.85787	0.297919	0.247395	0.017*	
C24	0.75004 (18)	0.07631 (17)	0.27528 (11)	0.0158 (2)	
C25	0.67098 (19)	−0.01344 (17)	0.33888 (11)	0.0175 (2)	
H25	0.635444	−0.126036	0.327557	0.021*	
C26	0.64509 (19)	0.06105 (16)	0.41707 (11)	0.0168 (2)	
H26	0.592223	−0.001036	0.459596	0.02*	
C101	1.2835 (2)	0.7674 (2)	1.04820 (14)	0.0279 (3)	
H10A	1.320626	0.688278	1.021153	0.042*	
H10B	1.303048	0.771121	1.118552	0.042*	
H10C	1.356958	0.873448	1.038389	0.042*	
C102	1.0013 (2)	0.8037 (2)	1.04333 (14)	0.0300 (4)	
H10D	1.008633	0.783988	1.109651	0.045*	
H10E	0.872272	0.762924	1.00362	0.045*	
H10F	1.062839	0.91934	1.0469	0.045*	
N10	1.09055 (17)	0.72197 (16)	0.99855 (10)	0.0211 (2)	
C10	0.9998 (2)	0.6113 (2)	0.91592 (12)	0.0240 (3)	
H10	0.873871	0.587458	0.88718	0.029*	
O10	1.06542 (18)	0.53699 (16)	0.87336 (9)	0.0302 (3)	
O1	0.8463 (2)	0.27288 (17)	0.72054 (11)	0.0263 (2)	
H4A	1.011 (3)	0.919 (3)	0.4152 (17)	0.032 (6)*	
H4B	1.005 (3)	0.780 (3)	0.3645 (18)	0.036 (6)*	
H1A	0.773 (4)	0.281 (3)	0.686 (2)	0.054 (9)*	
H1B	0.911 (4)	0.349 (3)	0.764 (2)	0.044 (7)*	

### Atomic displacement parameters (Å^2^)

**Table:**

	*U*^11^	*U*^22^	*U*^33^	*U*^12^	*U*^13^	*U*^23^
Ni1	0.01357 (7)	0.00904 (7)	0.01292 (8)	0.00464 (6)	0.00502 (6)	0.00230 (6)
Br1	0.02594 (7)	0.01691 (7)	0.02208 (8)	0.00886 (6)	0.01230 (6)	0.00042 (5)
C11	0.0134 (5)	0.0148 (6)	0.0142 (6)	0.0052 (4)	0.0043 (4)	0.0024 (5)
O11	0.0216 (4)	0.0124 (4)	0.0166 (5)	0.0069 (4)	0.0091 (4)	0.0027 (4)
C12	0.0136 (5)	0.0148 (6)	0.0149 (6)	0.0060 (4)	0.0047 (4)	0.0015 (5)
C13	0.0187 (6)	0.0165 (6)	0.0188 (6)	0.0076 (5)	0.0053 (5)	−0.0003 (5)
C14	0.0210 (6)	0.0230 (7)	0.0184 (6)	0.0101 (5)	0.0084 (5)	0.0009 (5)
C15	0.0202 (6)	0.0242 (7)	0.0171 (6)	0.0079 (5)	0.0085 (5)	0.0036 (5)
C16	0.0200 (6)	0.0163 (6)	0.0175 (6)	0.0056 (5)	0.0079 (5)	0.0031 (5)
C1	0.0165 (5)	0.0121 (5)	0.0165 (6)	0.0063 (4)	0.0053 (4)	0.0016 (4)
N2	0.0150 (4)	0.0106 (5)	0.0149 (5)	0.0049 (4)	0.0050 (4)	0.0033 (4)
N3	0.0197 (5)	0.0099 (5)	0.0172 (5)	0.0050 (4)	0.0077 (4)	0.0034 (4)
C4	0.0156 (5)	0.0099 (5)	0.0160 (6)	0.0045 (4)	0.0043 (4)	0.0032 (4)
N4	0.0253 (6)	0.0099 (5)	0.0201 (6)	0.0059 (4)	0.0119 (5)	0.0046 (4)
N5	0.0138 (4)	0.0089 (4)	0.0143 (5)	0.0043 (4)	0.0040 (4)	0.0026 (4)
C6	0.0140 (5)	0.0115 (5)	0.0145 (5)	0.0046 (4)	0.0053 (4)	0.0033 (4)
C21	0.0137 (5)	0.0118 (5)	0.0156 (6)	0.0054 (4)	0.0054 (4)	0.0027 (4)
O21	0.0204 (4)	0.0108 (4)	0.0181 (5)	0.0066 (3)	0.0102 (4)	0.0033 (3)
C22	0.0128 (5)	0.0107 (5)	0.0147 (5)	0.0042 (4)	0.0046 (4)	0.0028 (4)
C23	0.0153 (5)	0.0128 (5)	0.0160 (6)	0.0055 (4)	0.0067 (4)	0.0024 (5)
C24	0.0162 (5)	0.0147 (6)	0.0171 (6)	0.0067 (5)	0.0064 (5)	0.0010 (5)
C25	0.0201 (6)	0.0112 (5)	0.0231 (7)	0.0066 (5)	0.0101 (5)	0.0023 (5)
C26	0.0193 (6)	0.0114 (5)	0.0223 (7)	0.0055 (5)	0.0116 (5)	0.0055 (5)
C101	0.0209 (7)	0.0330 (9)	0.0281 (8)	0.0103 (6)	0.0069 (6)	0.0046 (7)
C102	0.0314 (8)	0.0339 (9)	0.0292 (9)	0.0212 (7)	0.0060 (7)	0.0042 (7)
N10	0.0206 (5)	0.0228 (6)	0.0208 (6)	0.0097 (5)	0.0063 (5)	0.0062 (5)
C10	0.0248 (7)	0.0239 (7)	0.0210 (7)	0.0063 (6)	0.0083 (6)	0.0092 (6)
O10	0.0366 (6)	0.0287 (6)	0.0245 (6)	0.0094 (5)	0.0159 (5)	0.0038 (5)
O1	0.0294 (6)	0.0320 (7)	0.0224 (6)	0.0169 (6)	0.0092 (5)	0.0081 (5)

### Geometric parameters (Å, º)

**Table:**

Ni1—N2	1.8383 (11)	C6—C22	1.4176 (18)
Ni1—N5	1.8494 (11)	C6—H6	0.95
Ni1—O21	1.8515 (10)	C21—O21	1.3048 (16)
Ni1—O11	1.8562 (10)	C21—C26	1.4241 (19)
Br1—C24	1.9044 (14)	C21—C22	1.4255 (19)
C11—O11	1.3142 (17)	C22—C23	1.4208 (19)
C11—C16	1.415 (2)	C23—C24	1.3651 (19)
C11—C12	1.4228 (19)	C23—H23	0.95
C12—C13	1.413 (2)	C24—C25	1.407 (2)
C12—C1	1.433 (2)	C25—C26	1.370 (2)
C13—C14	1.379 (2)	C25—H25	0.95
C13—H13	0.95	C26—H26	0.95
C14—C15	1.398 (2)	C101—N10	1.452 (2)
C14—H14	0.95	C101—H10A	0.98
C15—C16	1.382 (2)	C101—H10B	0.98
C15—H15	0.95	C101—H10C	0.98
C16—H16	0.95	C102—N10	1.453 (2)
C1—N2	1.2947 (18)	C102—H10D	0.98
C1—H1	0.95	C102—H10E	0.98
N2—N3	1.3926 (16)	C102—H10F	0.98
N3—C4	1.3069 (18)	N10—C10	1.327 (2)
C4—N4	1.3423 (18)	C10—O10	1.233 (2)
C4—N5	1.4133 (17)	C10—H10	0.95
N4—H4A	0.85 (2)	O1—H1A	0.72 (3)
N4—H4B	0.81 (2)	O1—H1B	0.80 (3)
N5—C6	1.3095 (17)		
			
N2—Ni1—N5	83.08 (5)	N5—C6—C22	124.01 (12)
N2—Ni1—O21	177.80 (5)	N5—C6—H6	118
N5—Ni1—O21	95.25 (5)	C22—C6—H6	118
N2—Ni1—O11	95.35 (5)	O21—C21—C26	118.42 (12)
N5—Ni1—O11	178.29 (5)	O21—C21—C22	124.57 (12)
O21—Ni1—O11	86.30 (4)	C26—C21—C22	117.02 (12)
O11—C11—C16	119.03 (13)	C21—O21—Ni1	126.68 (9)
O11—C11—C12	124.05 (12)	C6—C22—C23	117.06 (12)
C16—C11—C12	116.91 (12)	C6—C22—C21	122.19 (12)
C11—O11—Ni1	126.59 (9)	C23—C22—C21	120.75 (12)
C13—C12—C11	120.07 (13)	C24—C23—C22	119.52 (13)
C13—C12—C1	117.68 (13)	C24—C23—H23	120.2
C11—C12—C1	122.24 (12)	C22—C23—H23	120.2
C14—C13—C12	121.51 (14)	C23—C24—C25	120.99 (13)
C14—C13—H13	119.2	C23—C24—Br1	119.43 (11)
C12—C13—H13	119.2	C25—C24—Br1	119.57 (10)
C13—C14—C15	118.70 (14)	C26—C25—C24	120.21 (13)
C13—C14—H14	120.7	C26—C25—H25	119.9
C15—C14—H14	120.7	C24—C25—H25	119.9
C16—C15—C14	120.97 (14)	C25—C26—C21	121.51 (13)
C16—C15—H15	119.5	C25—C26—H26	119.2
C14—C15—H15	119.5	C21—C26—H26	119.2
C15—C16—C11	121.82 (14)	N10—C101—H10A	109.5
C15—C16—H16	119.1	N10—C101—H10B	109.5
C11—C16—H16	119.1	H10A—C101—H10B	109.5
N2—C1—C12	123.84 (13)	N10—C101—H10C	109.5
N2—C1—H1	118.1	H10A—C101—H10C	109.5
C12—C1—H1	118.1	H10B—C101—H10C	109.5
C1—N2—N3	115.09 (12)	N10—C102—H10D	109.5
C1—N2—Ni1	127.91 (10)	N10—C102—H10E	109.5
N3—N2—Ni1	116.98 (9)	H10D—C102—H10E	109.5
C4—N3—N2	110.50 (11)	N10—C102—H10F	109.5
N3—C4—N4	120.38 (12)	H10D—C102—H10F	109.5
N3—C4—N5	117.40 (12)	H10E—C102—H10F	109.5
N4—C4—N5	122.21 (12)	C10—N10—C101	121.63 (14)
C4—N4—H4A	114.7 (15)	C10—N10—C102	121.41 (14)
C4—N4—H4B	123.3 (17)	C101—N10—C102	116.96 (14)
H4A—N4—H4B	115 (2)	O10—C10—N10	125.34 (16)
C6—N5—C4	120.76 (12)	O10—C10—H10	117.3
C6—N5—Ni1	127.20 (9)	N10—C10—H10	117.3
C4—N5—Ni1	112.02 (9)	H1A—O1—H1B	115 (3)
			
C16—C11—O11—Ni1	177.39 (10)	N3—C4—N5—Ni1	−0.36 (15)
C12—C11—O11—Ni1	−1.81 (19)	N4—C4—N5—Ni1	−178.86 (11)
N2—Ni1—O11—C11	1.06 (12)	N2—Ni1—N5—C6	−178.04 (12)
O21—Ni1—O11—C11	179.59 (11)	O21—Ni1—N5—C6	3.41 (12)
O11—C11—C12—C13	−179.83 (13)	N2—Ni1—N5—C4	0.59 (9)
C16—C11—C12—C13	0.96 (19)	O21—Ni1—N5—C4	−177.96 (9)
O11—C11—C12—C1	1.4 (2)	C4—N5—C6—C22	178.98 (12)
C16—C11—C12—C1	−177.81 (13)	Ni1—N5—C6—C22	−2.50 (19)
C11—C12—C13—C14	−0.3 (2)	C26—C21—O21—Ni1	−178.48 (9)
C1—C12—C13—C14	178.49 (13)	C22—C21—O21—Ni1	1.61 (19)
C12—C13—C14—C15	−0.9 (2)	N5—Ni1—O21—C21	−2.96 (11)
C13—C14—C15—C16	1.4 (2)	O11—Ni1—O21—C21	177.76 (11)
C14—C15—C16—C11	−0.8 (2)	N5—C6—C22—C23	179.74 (12)
O11—C11—C16—C15	−179.68 (13)	N5—C6—C22—C21	0.0 (2)
C12—C11—C16—C15	−0.4 (2)	O21—C21—C22—C6	0.4 (2)
C13—C12—C1—N2	−179.16 (13)	C26—C21—C22—C6	−179.48 (12)
C11—C12—C1—N2	−0.4 (2)	O21—C21—C22—C23	−179.27 (13)
C12—C1—N2—N3	−178.84 (12)	C26—C21—C22—C23	0.82 (18)
C12—C1—N2—Ni1	−0.2 (2)	C6—C22—C23—C24	−179.73 (12)
N5—Ni1—N2—C1	−179.40 (13)	C21—C22—C23—C24	−0.02 (19)
O11—Ni1—N2—C1	−0.08 (13)	C22—C23—C24—C25	−0.7 (2)
N5—Ni1—N2—N3	−0.76 (9)	C22—C23—C24—Br1	−179.66 (10)
O11—Ni1—N2—N3	178.56 (9)	C23—C24—C25—C26	0.5 (2)
C1—N2—N3—C4	179.55 (12)	Br1—C24—C25—C26	179.49 (11)
Ni1—N2—N3—C4	0.73 (14)	C24—C25—C26—C21	0.4 (2)
N2—N3—C4—N4	178.31 (12)	O21—C21—C26—C25	179.09 (13)
N2—N3—C4—N5	−0.22 (17)	C22—C21—C26—C25	−1.0 (2)
N3—C4—N5—C6	178.37 (12)	C101—N10—C10—O10	0.1 (3)
N4—C4—N5—C6	−0.1 (2)	C102—N10—C10—O10	179.88 (17)

### Hydrogen-bond geometry (Å, º)

**Table:**

*D*—H···*A*	*D*—H	H···*A*	*D*···*A*	*D*—H···*A*
N4—H4*A*···N3^i^	0.85 (2)	2.16 (2)	3.0116 (17)	176 (2)
N4—H4*B*···O1^ii^	0.81 (2)	2.09 (2)	2.8900 (19)	169 (2)
O1—H1*A*···O11	0.72 (3)	2.38 (3)	3.0056 (17)	146 (3)
O1—H1*A*···O21	0.72 (3)	2.48 (3)	3.0719 (18)	141 (3)
O1—H1*B*···O10	0.80 (3)	1.97 (3)	2.772 (2)	178 (3)

Symmetry codes: (i) −*x*+2, −*y*+2, −*z*+1; (ii) −*x*+2, −*y*+1, −*z*+1.
